# A comparison of high-throughput techniques for assaying circadian rhythms in plants

**DOI:** 10.1186/s13007-015-0071-9

**Published:** 2015-05-03

**Authors:** Andrew J Tindall, Jade Waller, Mark Greenwood, Peter D Gould, James Hartwell, Anthony Hall

**Affiliations:** Institute of Integrative Biology, University of Liverpool, Crown Street, Liverpool, UK

**Keywords:** Luciferase, Leaf movement, Delayed fluorescence, Infra-red gas exchange, Circadian clock, High-throughput assay

## Abstract

Over the last two decades, the development of high-throughput techniques has enabled us to probe the plant circadian clock, a key coordinator of vital biological processes, in ways previously impossible. With the circadian clock increasingly implicated in key fitness and signalling pathways, this has opened up new avenues for understanding plant development and signalling. Our tool-kit has been constantly improving through continual development and novel techniques that increase throughput, reduce costs and allow higher resolution on the cellular and subcellular levels. With circadian assays becoming more accessible and relevant than ever to researchers, in this paper we offer a review of the techniques currently available before considering the horizons in circadian investigation at ever higher throughputs and resolutions.

## Introduction to the plant circadian clock

Circadian clocks are endogenous, persistent, temperature-compensating timekeepers which provide temporal organization of biological processes from cyanobacteria to man [1]. In plants, circadian rhythmicity is widespread; a transcriptional circadian cycling has been reported in a range of diverse species [2], including the model plant *Arabidopsis thaliana* in which the clock has been well characterized [3]. Approximately one-third of the *Arabidopsis* transcriptome shows circadian oscillations in abundance when in free-run conditions [4], indicating direct or indirect circadian control.

It is becoming increasingly clear that robust circadian rhythms are integral to overall fitness [3], are key players in the control of flowering time [5], are regulators of the susceptibility and response to pathogen attack [6-8], and are linked to important agronomic traits in multiple crop species including potato, rice, wheat, barley and soybean [9-15].

In the last twenty years, we have begun to elucidate essential features of the clock in the model plant Arabidopsis thaliana. The clock appears thus far to be considerably conserved with the rest of the plant kingdom [3,16,17]. Thanks to molecular studies, and the use of circadian reporter systems, we know that the central oscillator consists of three interlocking auto-regulatory transcriptional feedback loops. LATE ELONGATED HYPOCOTYL 1 (LHY) and CIRCADIAN CLOCK ASSOCIATED 1 (CCA1) repress transcription of their inducer *TIMING OF CAB EXPRESSION 1 (TOC1)* at dawn, forming the “morning loop” [18]. A second “evening loop”, comprising of GIGANTEA (GI) as an inducer of *TOC1* transcription which is in turn repressed by CCA1/LHY1, was predicted in silico to fit empirical observations [19] and has had it’s existence confirmed experimentally [20]. The final transcriptional loop represses transcription of *CCA1/LHY* through the activity of PSEUDO-RESPONSE REGULATOR 7 (PRR7) and PRR9 and is known as the “Night Inhibitor” loop (NI) [21]. This transcriptional-level model is further augmented by post-transcriptional and post-translational modifications that affect clock function. GI protein regulates the evening loop at the post- translational level through the stabilisation of the F-box protein ZEITLUPE (ZTL) in the presence of light [22] which, in turn, is necessary for the targeting of TOC1 protein for degradation by the proteasome. The connections between LHY and the night inhibitor loop [23], as well as between TOC1 and CCA1/LHY [24], are also post-translational in nature.

Our understanding of the clock, however, is far from complete. Environmental inputs and outputs of the clock are not well characterised. Whilst the transcription of a significant number of genes appears to be circadian regulated, relatively few possess a predicted circadian motif in their promoter, suggesting control through as-yet-to-be implicated factors [25,26]. Additionally, whilst it is known that the central transcriptional clock is tuned or “entrained” by diurnally oscillating external stimuli such as red and blue light [27,28], temperature [29], and phytohormones [30], it remains unclear how these pathways are regulated.

In the last two decades, our knowledge of the plant circadian clock has been vastly improved through the development of new assay techniques. The high-throughput nature of these assays has led us towards a more integrated understanding of how the circadian system works and, more over, has allowed us to perform work that would previously be impossible. In this review, we outline the major techniques that have been developed for investigating the circadian clock in plants before considering the possible horizons that will open up following future assay development.

## The need for high-throughput assays in circadian investigation

The circadian system can be probed in considerable depth through the use of molecular techniques such as quantitative-PCR and Northern blotting to assay clock gene expression directly over a sampling time-course. Indeed, these techniques are used extensively in circadian studies alongside high-throughput techniques, having been used to characterise numerous clock components, most notably *CCA1* [31] and *LHY* [32]. More recently, the proliferation of micro-arrays and RNA-seq technology has expanded the molecular toolkit, allowing us to view the abundance and splicing patterns of multiple mRNAs simultaneously [33,34]. They are still vital tools for in-depth characterisation and investigation of the mechanism of action for individual circadian genes, and for teasing apart the roles of separate clock loops.

However, molecular techniques are considerably time and resource consuming, requiring the researcher to perform multiple, regular samplings over several days. They also require destructive sampling – the harvest of leaf tissue, whole plants or groups of plants– which not only requires lots of plant growth space, but involves large amounts of resource-consuming wet-work at the bench following the time-course. This necessarily reduces the resolution and throughput of the assay, limiting not only the sampling frequency but also the number of parallel and consecutive experiments that the lab’s finances (and level of fatigue) can support. Additionally, destructive sampling raises issues with averaging biological variation between tissues as well as between plants, making observation of an individual plant or tissue throughout a time-course impossible. Together, this makes molecular techniques unsuitable for the wide-scale screening and initial identification of clock genes.

By means of contrast, high throughput assays are far better suited to screening. As non-invasive, non-destructive techniques they allow concurrent sampling on the same plant without perturbing the clock through stress or killing it. They also allow a high level of automation. As opposed to physical harvest, the assays outlined below can be set up and left to run with minimal human intervention, compared with methods where harvest have to be performed regularly over several days. This has resulted in a paradigm shift in recent years, whereby the limiting factor in the throughput of circadian investigations has become the data handling and preparation of plant materials, and the scale of these experiments is limited only by the capacity and number of assay machines. This allows researchers to carry out broader, further-reaching experiments investigating the influence of several factors on the clock to identify candidates for future in-depth study.

## Lumino-fluorescent circadian reporters

### Transgenic luciferase as a circadian reporter

Increasing numbers of investigations of the transcriptional circadian oscillator are using transgenic luciferase reporters to probe clock period and robustness [35,36]. Luciferase is an enzyme that catalyses the light-emitting ATP-dependent monooxidation of luciferin, a compound with which test plants are dosed. Typically, the brighter, modified *LUC+* gene sourced from the firefly *Photinus pyralis* is used [37]. Luminescence intensity can be recorded through the use of photo-multiplier tubes attached to detectors, as in the TopCount system, or, increasingly, high sensitivity charge-coupled cameras enclosed within light-tight growth chambers with automated lights [36].

In a typical cell, following saturation with luciferin, oxygen and ATP are in excess; as such the amount of luminescence observed is directly dependent on how much luciferase is present [35]. As luciferase is an unstable enzyme that rapidly loses function, the amount of functional luciferase is directly determined by the rate of luciferase expression. When under the control of a circadian-regulated promoter, the observed luminescence provides a quantitative measure of promoter-driven gene expression. This makes luciferase, when placed under the control of a circadian-regulated promoter such as *CAB2* or *CCR2* [38], an excellent reporter of clock transcriptional output [35,39,40]. This technique has been used to identify several core clock genes, most notably *TOC1* [41], and to investigate the roles external entraining stimuli such as temperature [42-44] and sucrose [45] play in clock function.

Interestingly, in recent years the sensitivity of luminescence imaging cameras has increased to the point where more specific resolution is now possible. In the duckweed *Lemna gibba*, circadian rhythms in *AtCCA1:LUC+* have been detected within individual cells following transient transformation by particle bombardment [46]. In *Arabidopsis*, tissue-specific clocks have been identified in the leaf mesophyll and vasculature through the use of split-luciferase assays, wherein half of the luciferase protein is driven by a clock promoter and another half by a tissue-specific promoter, which are only luminous when expressed together in the same cell [47]. However, whilst luciferase has been used successfully to assay clock function in various species, most notably tobacco, arabidopsis [35] and rice [48], the need to introduce transgenic luciferase into the plants is a time-consuming step that greatly reduces throughput and renders the system unsuitable for species without a transformation protocol. Whilst currently transgene expression is dependent on promoter activity and the genomic context of the random transgenic insertion, in the future and through the use of precision genome engineering techniques [49] we are likely to see luciferase being fused directly to endogenous genes within the native context.

### Delayed fluorescence as an endogenous, universal assay

First described in 1951 [50], delayed fluorescence is the emission of light from plants, algae and cyanobacteria following their transfer from light to dark conditions resulting from charge recombination in the photosynthetic machinery [51], primarily within the P680 light harvesting complex of photosystem II [52-54].

In normal photosynthesis, incoming photons excite electron pairs in the light harvesting complexes of the photosystems, raising them to higher energy states. These electrons are passed to receiver molecules further down the photorespiratory chain which use their stored energy to generate a membrane potential, pump protons across the thylakoid membrane, and do biological work [55]. Charge-recombination between the receiver molecule plastoquinone QA re-excites the P680 complex, which produces fluorescence as it returns to the ground state through the release of stored energy in the form of a photon [53]. Approximately 0.03% of absorbed solar energy is re-emitted in this manner [53]. The intensity of the delayed fluorescence emission decays rapidly to undetectable near-background levels over the course of a minute [56,57]; it is therefore critical that images are taken over exactly the same period following lights off in order to observe rhythmicity. This persistence allows the long exposure windows required for detailed imaging. However, because the intensity decays exponentially and rapidly, accurate and precise control of the light source and length of delay is required to acquire accurate data.

The intensity of delayed fluorescence has been shown to be under the control of the circadian clock. The periods and robustness of known clock mutants *lhy-21*, *cca1-11*, *gi-11* and *toc1-2* as detected by delayed fluorescence agree with those previously determined by other techniques [58]. Whilst the exact nature of the relationship between the clock and delayed fluorescence is still unclear, many of the key genes that make up the light harvesting complexes within PSI and II are under circadian control at the transcriptional level [59], providing a possible link between the nuclear transcriptional clock and this particular output.

As a platform for circadian phenotyping, delayed fluorescence provides a non-transgenic system that can be used to assay the clock in a vast number of species. The CCD cameras used for luciferase imaging are sufficiently sensitive for delayed fluorescence assays, provided that the growth chamber and lights are under accurate precision control [58]. To date, it has been used to investigate plant species including *Arabidopsis*, the C3 monocots barley [58] and einkorn wheat [60], the C4 monocot maize [58], the model CAM species *K. fedtschenkoi* [58], and the coniferous gymnosperm Norway spruce (*Picea abies*) [61]. The system provides a quick and simple, non-invasive, universal platform for phenotyping plant clocks across species and taxa without the need for transformation.

## Leaf movement as a circadian reporter

Along with diel rhythms in stem and root elongation, leaf movement has long been well-characterised as a growth-dependent circadian clock output [62]. Circadian-regulated oscillations in leaf position are a result of differential patterns of growth in cells on opposing adaxial and abaxial sides of the petiole (the structure that connects the leaf blade to the stem, which causes the positions of young leaves to rise and fall throughout the 24 hour period [63,64]. This movement is differentially phased from petiole elongation, and requires a functional *ELF3* and evening complex for proper phasing [64].

Although the biological importance of leaf movement remains unproven, it has long been used as a circadian reporter. The plant clock was first described through the study of rhythmic pulvinus-driven opening and closing of leaves in *Mimosa pudica* [65]. Low-throughput leaf movement assays have also provided insight into the clock in *Arabidopsis*, with the absence of leaf movement rhythms in *lhy* mutants helping to implicate that gene in the circadian system [32]. Measuring directly the clock controlled physiological outputs such as leaf movement provides a non-invasive assay that, unlike luciferase reporters, does not require transformation. Leaf movement is favoured over other physiological outputs due to the increased rhythm robustness and ease of assay [66]. Computer-automated image capture by CCD within the growth chamber can be performed throughout the time-course [67]. Over the past decade several straight-forward systems have been developed based on computer-automated capture of leaf position [68], whilst advancements in digital camera technology has reached a point where consumer-level cameras are suitable for circadian imaging, dramatically driving down the hardware costs of such systems [69]. Compared to lumino-fluorescent techniques, with associated equipment costs in the tens of thousands of pounds, relatively cheap digital cameras have removed a major barrier to entry for circadian phenotyping. The more recent development of sophisticated machine vision algorithms and reliable computer automation have helped automate the leaf tracking analysis, previously a time-consuming bottleneck in the pipeline for the researcher. Together this has gone a long way to enabling the use of leaf movement as a high throughput clock assay.

Whilst this system can be used in non-transformable plants, it cannot be used for species with sessile leaves (i.e. those that lack petioles). This makes the vast majority of monocots, including all major cereal crops, unsuitable for this technique. Furthermore, in *Arabidopsis*, leaf movements halt once the leaves are mature which equates to a window for assay of approximately one week [67]. Regardless, leaf movement is a robust and accurate assay that, with further development and ever-reducing hardware costs, is an attractive and valuable tool for investigating circadian function. With the advent of sophisticated image analysis algorithms, in the future other circadian regulated growth processes are likely to be developed into high throughput systems, for example hypocotyl growth [66].

## Infra-red gas exchange

Observations from various studies throughout the past century have alluded to the fact that carbon dioxide fixation displays a 24-hour rhythm in photosynthetic organisms [70] and this has long since been shown to be a reliable measure of circadian output [71,72]. Carbon fixation can be assayed quickly through use of an Infra-Red Gas Exchange Analyser (IRGA), whereby whole or partial plants are grown in chambers containing a known, controlled atmosphere, and the resulting output air analysed for it’s composition, allowing photosynthetic rate to be defined rapidly and with high resolution, with sample readings being taken multiple times per hour.

The IRGA has become an effective tool to assay the circadian clock, especially within the crassulacean acid metabolism community [73] where it has proven useful at identifying peripheral and partially-redundant components that when perturbed feedback to affect the clock which have otherwise proved elusive [74]. The technique has also been used to analyse circadian rhythms in stomatal conductance in the *toc1-1* mutant of *Arabidopsis thaliana* [75]. As carbon fixation is universally clock-regulated in plants, this technique is applicable to many plant species.

Whilst the IRGA system is becoming more practical, with a range of models in production including large multichannel machines that allow parallel analysis and portable field versions, and flexible, with the sealed chambers allowing manipulation and comparison of multiple environmental conditions, it is not without drawbacks. As with any indirect assay, it allows a gauge of overall circadian health, rather than specific investigation of particular clock loops. The level of clock control on net carbon fixation is complex, with contributions at the level of photosynthetic gene expression all the way up to regulation of stomatal conductance and growth. None-the-less it allows investigation of the plant clock on a level that is otherwise unaccounted for by other techniques.

## Data analysis solutions

With the development of high-throughput assays, developing large data-sets in ever decreasing time frames, data analysis and period identification has increasingly become a time-consuming and rate-reducing step in circadian investigation. Fortunately, new methods for estimating the underlying period have been developed that can reduce and simplify this process. With a variety of different techniques and algorithms of varying complexity, choosing which is most applicable can prove challenging.

The related curve-fitting algorithms mFourfit [76] and Fast Fourier Transform Non-Linear Least Squares (FFT-NLLS) [77,78] have long been central period estimators in circadian investigations. FFT-NLLS has the additional advantage of providing the confidence intervals for the predicted periods, phases and amplitudes, allowing us to identify arrhythmic samples in a manner that mFourfit cannot. It does, however, tend to “over-fit” the series and, as such, is poor at identifying noise in the data.

Curve-fitting is not the only technique for analysis - stochastic modelling approaches can be used to estimate periods, as in Maximum Entropy Spectral Analysis (MESA) [79]. This performs better when identifying periods for datasets with large baseline trends. An additional technique, Spectrum Resampling (SR), is based around iterative boot-strapping of a smoothed power series to provide a steady-state model which reflects the data. Whilst MESA and SR especially are designed to be more robust when confronted with observational noise [80], they are considerably more computationally taxing than other methods.

We recommend, if possible, performing simultaneous analysis with techniques from both curve-fitting and stochastic modelling schools for analysing data. As all of these techniques are available through the BioDARE service, hosted by the University of Edinburgh [81], it is relatively painless to perform multiple analyses to gather accurate period estimations and simply share data in a standard format following publication.

## Emerging assays

So far, the vast majority of high-throughput circadian assays have looked at the plant group in groups of seedlings, whole-plant or whole leaf scale (Table 1). Recently, novel circadian assays have been developed that allow the clock to be surveyed within individual tissues, individual cells and beyond to the sub-cellular level. In addition to the single-cell and tissue-specific luciferase based systems outlined above, fluorescence based techniques have potential as circadian assays [82]. This has been done successfully using fluorescent protein tagged CCA1 as a marker to probe the intracellular dynamics of CCA1 [83] identifying the independence of the guard cell clock from that of the surrounding leaf [84]. Although this paper has made significant strides in measuring the clock at the single cell level, major bottlenecks with this type of technique still exist. To make single cell circadian research plausible, new techniques need to be identified that allow oscillations to be measured for several days in different tissue types across hundreds of cells. Further development and adoption of these and related techniques will bring a new fine-scale understanding of the circadian clock.

**Table 1:** **Summary comparison of circadian screening techniques**

**Assay**	**Type**	**Species suitability**	**Requires**	**Max resolution**	**Throughput**	**Ref.**
			**transformation?**			
Western Blot/qPCR	Direct assay of	All plants	No	Single Cell/Tissue	Low	[89]
	gene expression.					
Luciferase	Direct assay of	Transformable plants	Yes	Single Cell/Tissue	High	[36]
	gene transcription.					
Delayed fluorescence	Indirect assay of	Most plants	No	Whole plant	High	[58]
	clock phenotype.					
Leaf movement	Indirect assay of	Most Dicots	No	Whole plant	High	[67,69]
	clock phenotype.					
IRGA	Indirect assay of	All plants	No	Whole organ (Individual leaves)	Medium	[73]
	clock phenotype					

Contrary to previous belief that the clock was cell autonomous [85], increasing amounts of evidence has emerged to suggest inter cellular communication between circadian clocks. Weak communication between individual circadian clocks has been observed in between shoots and roots [86] and within leaves [87]. Comparative assay of clock-driven luciferase expression in roots and leaves has provided evidence of phased “waves” of coordination through the tissue from root to shoot [88]. Using tissue-specific luciferase assays, it has demonstrated that there is significant communication between the vascular and mesophyll clocks, with the vascular clock serving as the dominant coordinator [47]. The required resolution of these techniques is currently on the threshold of the detection limit. It seems likely, in light of these discoveries, that further inter-cellular coordination will be uncovered and prove instrumental in understanding the clock system in the wider context of the organism in the environment.

## Conclusion

For the first time, we are equipped with the tools to rapidly, accurately and extensively investigate the clock architecture in ways that go beyond it’s temporal arrangement. We are also capable of assaying various clock outputs and reporters in different tissues. With the development of higher-resolution techniques and more powerful imaging methods, our understanding of the clock and it’s roles will expand to incorporate different spacio-developmental contexts, and approach a fuller picture of underlying circadian coordination in plants.
